# Potential Antiviral Activity of *Lactiplantibacillus plantarum* KAU007 against Influenza Virus H1N1

**DOI:** 10.3390/vaccines10030456

**Published:** 2022-03-16

**Authors:** Irfan A. Rather, Majid Rasool Kamli, Jamal S. M. Sabir, Bilal Ahmad Paray

**Affiliations:** 1 Department of Biological Sciences, Faculty of Science, King Abdulaziz University, P.O. Box 80203, Jeddah 21589, Saudi Arabia; mkamli@kau.edu.sa (M.R.K.); jsabir@kau.edu.sa (J.S.M.S.); 2 Center of Excellence in Bionanoscience Research, King Abdulaziz University, P.O. Box 80203, Jeddah 21589, Saudi Arabia; 3 Department of Zoology, College of Science, King Saud University, P.O. Box 2455, Riyadh 11451, Saudi Arabia; bparay@ksu.edu.sa

**Keywords:** camel milk, lactic acid bacteria, probiotics, H1N1, influenza, *Lactiplantibacillus plantarum*

## Abstract

The development of antiviral resistance has exacerbated a growing threat to public health. As a result, there is increasing demand for unconventional antivirals that can effectively replace the presently in-use drugs. Lactic acid-producing bacteria (LAB) are among the most common bacteria used in the food industry. These bacteria play an essential role in the fermentation of many foods and feed. Additionally, these bacteria are considered more economical, efficient, and safe “nutraceuticals” in the health care arsenal. Therefore, we carried out the screening and molecular characterization of raw camel milk LAB isolates and tested their inhibitory activity against influenza virus H1N1. The strain that exhibited the highest antiviral activity against the H1N1 virus, confirmed by hemagglutination assay, was identified as *Lactiplantibacillus plantarum* KAU007. The study also confirmed the non-cytotoxic behavior of CFCS isolated from KAU007 against MDCK cells, approving its safety concern against the mammalian cells. Besides, CFCS at 5 and 10 mg/mL significantly decreased the level of IFN-γ (*p* < 0.001 and *p* < 0.001) and IL-6 (*p* < 0.001 and *p* < 0.005) in a dose-dependent manner, respectively. This is a preliminary report about the anti-influenza activity of KAU007 isolated from camel milk. This study reinforces that camel milk contains beneficial LAB isolates with antagonistic properties against the H1N1 influenza virus.

## 1. Introduction

“Flu” is a contagious respiratory sickness caused by influenza viruses. A flu epidemic can cause 3 to 5 million severe illnesses and 250,000 to 500,000 influenza-related death worldwide [1,2]. Despite the viability of influenza vaccines, influenza results in approximately 36,000 deaths and up to 220,000 hospitalizations in the United States alone [3]. This can be partly attributed to the fact that current vaccination programs either have limited efficacy or are effective only when vaccine strains match circulating influenza viruses and antiviral drugs are limited in their ability to combat influenza [4]. The H1N1 pandemic virus in 2009 illustrated the inefficacy of current vaccines [5,6]. A total of 18 different HA (H1–H18) and H11 different NA subtypes (N1–N11) have been identified so far, which suggests that there are many different HA and NA combinations [7]. These strain-specific changes make it impossible to develop the vaccine for all. Therefore, to provide frontline protection against influenza viruses, it is necessary to find alternative means of prevention regardless of strain specificity. Besides, there is a growing demand for unconventional measures to protect against the disease [5,8,9], and new variants of these viruses have become a global healthcare threat [10,11,12].

Lactic acid bacteria are the most common probiotics that confer health benefits on their hosts. Therefore, probiotics are live microorganisms that benefit the host, similar to the gut microflora [4,13,14,15,16,17,18,19,20,21,22], and are known to survive in gastric and bile acid in the gastrointestinal tract [23,24]. Various probiotic LAB strains have been reported in fermented foods and dairy products [1,2,14,16,17,23,24,25,26,27,28,29,30,31,32]. However, LABs from camel milk are less explored for their health benefits. In mice [2,33], chicken [25], and humans [2,34], LAB probiotic strains have shown significant protection against a various number of infectious diseases [17,19,20,21,25,35,36,37,38,39,40,41,42]. Intranasal and oral pretreatment with LAB strains proved effective in protecting mice against influenza virus infection but did not protect against severe weight loss disease [43,44]. Furthermore, various trials have shown that preventing a cold in healthy elders and extending the survival time for mice with influenza has shown positive results [2,19,35,45]. Many studies found that some novel probiotic isolates increase innate and acquired immunity [46,47,48], increasing antiviral defenses [25,43,44,46].

Of note, it remains unclear whether treatment with LAB could provide some degree of improved protection. Therefore, this study was performed to visualize the antiviral effect of a probiotic strain *Lactiplantibacillus plantarum* KAU007 (*L. plantarum* KAU007) against the H1N1 influenza virus on MDCK mammalian cells.

## 2. Materials and Methods

### 2.1. Isolation and Identification of Probiotics from Camel Milk Samples

A total of 10 healthy camels were selected randomly to collect raw milk in Jeddah, Saudi Arabia. Fresh samples of milk were collected directly from the camel’s udder. The udder was cleaned with autoclaved distilled water and dried using a single-service towel. Three initial streams of milk were discarded before being collected for testing. After being collected into sterile tubes, the milk samples were kept in an icebox and then transported to the laboratory within 2 h.

Further, samples were diluted (1–10) to isolate the beneficial microorganisms from the milk. A homogenized solution of 10 mL of camel milk and 90 mL of saline H_2_O_2_ was prepared for this purpose. On de Man Rogosa (MRS) agar plates, 1 mL of each dilution was plated and incubated for 24 to 48 h. Several colonies were selected phenotypically and subsequently sub-cultured in PCB media to confirm lactic acid bacteria. Finally, chosen isolates were sent for 16S rRNA gene sequencing (Macrogen, Seoul, Korea). The selected isolates were stored at −80 °C before being used in in vitro experiments. Each isolate was subsequently sub-cultured two times in MRS broth for 18 to 24 h at 37 °C, supported by 20% (*v*/*v*) glycerol, and was activated before being used for experiments by sub-culturing three times for 48 h at 37 °C.

### 2.2. Preparation of Cell-Free Culture Supernatant

For cell-free culture supernatant (CFCS) preparation, the isolate was grown in MRS media for 18 h at 37 °C. Briefly, the LAB culture was collected and subjected to centrifugation for 10 min at 10,000 rpm at 4 °C. Then, the supernatant was carefully collected in a 50 mL syringe and filter-sterilized using 0.22 μm syringe filters. The filtered supernatant was neutralized (pH 6.5) with 1 M NaOH, followed by lyophilization. The sample was stored at 4 °C until used for further studies.

### 2.3. Mammalian Cell Culture and Its Maintenance

The MDCK cell line was routinely cultured in the laboratory by the same method described in our previously published reports [12,26,46]. Briefly, the cells were cultured in DMEM, supplemented with 10% FBS and 0.1% antibiotic/antimycotic solution, and then maintained in 5% CO_2_ and 95% air at 37 °C. All the in vitro assays were performed at 70–80% cell density.

### 2.4. Cytotoxicity Analysis of CFCS

To evaluate the cytotoxicity of CFCS, the MDCK cells were treated for 24 h in a dose-dependent manner (0–10 mg/mL). Specifically, the cells were plated at a density of 2 × 10^4^ in 96 well plates and allowed to proliferate until they reached 80% confluency. After the cells reached 80% confluency, the cells were treated with CFCS with various concentrations (2.5, 5, and 10 mg/mL) to evaluate its cytotoxicity against the mammalian cells. For the control, only PBS was used. After 24 h of treatment, the cell culture medium was aspirated from the wells, and 100 μL of fresh recommended culture medium was added into each well with 10 μL of MTT (5 mg/mL) per well, followed by incubation at 37 °C for 4 h. In the following step, the culture medium was enunciated out. Then the insoluble emerging formazan crystals were dissolved in 50 μL DMSO and further incubated at room temperature for 30 min in a well plate shaker. A microplate reader was used for measuring the absorbance at 540 nm. The percentage of viable cells was estimated using the following formula:

$$\mathrm{Cell}\;\mathrm{viability}\left(\%\right)=\frac{\;\mathrm{the}\;\mathrm{optical}\;\mathrm{density}\;\mathrm{of}\;\mathrm{the}\;\mathrm{treated}\;\mathrm{cells}-\mathrm{optical}\;\mathrm{density}\;\mathrm{of}\;\mathrm{control}}{\mathrm{optical}\;\mathrm{density}\;\mathrm{of}\;\mathrm{control}}\;\times 100$$

### 2.5. Antiviral Test of CFCS against MDCK Cells

Influenza virus H1N1 (A/Korea/01/2009) was previously obtained from KCDA, Korea. The viral strain was propagated in the MDCK cell model for 72 h under 4% CO_2_ environment at 37 °C. Next, 5-min centrifugation at 1500 rpm was carried out to isolate the virus from the infected MDCK cell. Our previous work describes the H1N1 titer and culture in detail [16,25,39]. H1N1 virus was treated with CFCS in concentrations of 2.5, 5 and 10 mg/mL, under 5% CO_2_, for 1 h at 37 °C. It was then injected into MDCK cells and cultured at 37 °C in a humidified chamber with 5% CO_2_ for two days. The cells were plated onto DMEM plates containing 2% FBS, 100 g/mL streptomycin and 1% (*v*/*v*) 100 U/mol penicillin. The plates were observed for cytopathic effect (CPE) after 2 days. The absence of CPE in plates was considered as the presence of antiviral activity [25].

### 2.6. Hemagglutination Inhibition

Hemagglutination titrations of H1N1 strains were determined using a standard method [49] and our previously described method [25]. The CFCS of 2.5, 5, and 10 mg/mL) was used together with H1N1 (10^6.5^ EID_50_/0.1 mL) for injection into the allantoic cavity of 11 days old specific pathogen-free (SPF) embryonated eggs as per our previously used method [25,39].

### 2.7. Cytokine Profile

Cells were cultured in the recommended media until they reached 80% cell density. After that, cells were treated with CFCS at various concentrations (2.5, 5, and 10 mg/mL) and incubated for 24 h. The levels of interleukins IFN-γ and IL-6 were estimated using commercially available quantitative sandwich ELISA kits (R&D Systems, Inc, Minneapolis, MN, USA), according to the manufacturer’s protocol. A 450 nm wavelength was used to read the plates. The results were expressed as milligrams of protein per plate.

### 2.8. Statistical Analysis

This study’s experimental results were expressed as a mean x standard deviation (SD) to determine the quantitative results. To determine the statistical significance and the differences among the experimental groups, we used one-way analysis of variance (ANOVA) with Tukey’s test for comparing each pair of columns and Dunnett’s post-comparison test for comparing multiple groups, where * represents *p*-values < 0.05, ** represents *p*-values < 0.01, *** represents *p*-values < 0.001, and ns represents non-significant. The graphs were prepared using Prism 9.

## 3. Results and Discussion

### 3.1. Isolation, Purification, and Identification of Probiotic LAB Isolate

A total of 10 strains were isolated from the camel milk. After preliminary screening of antiviral activity, strain KAU007 showed the highest inhibition activity against H1N1. The strain was sent for 16S rRNA gene sequencing and was identified as *L. plantarum* (GenBank accession no. OM442911), hereafter named as *L. plantarum* KAU007.

### 3.2. Cytotoxic Assessment of CFCS against MDCK Cells

**Figure 1:**
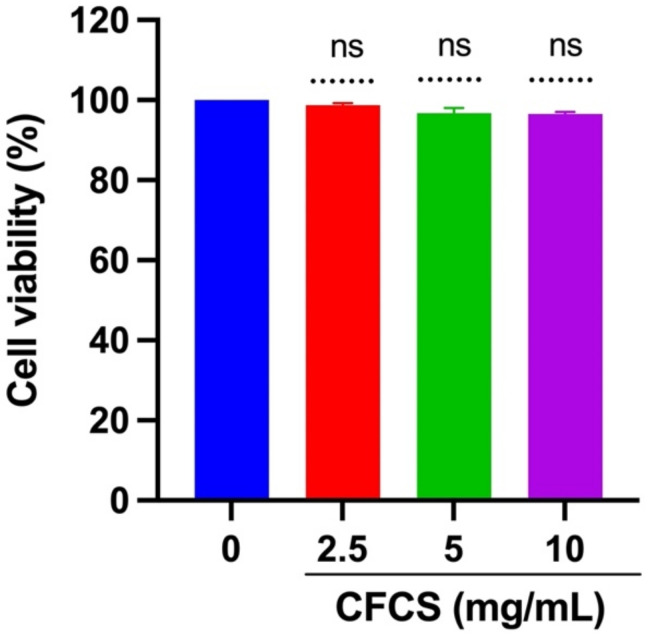
Cytotoxicity of CFCS in mammalian cells. The percentage of cell viability was determined using an MTT assay. The experiments were performed in triplicate, and the results were presented as mean ± SE values; ns = no statistically significant difference between the groups.

The MTT assay was used to determine the cell viability of MDCK cells treated with CFCS to assess the cytotoxicity of CFCS within the host body. It can be seen from Figure 1 that the chosen concentrations (2.5, 5, and 10 mg/mL) did not have significant effects on the MDCK cells when compared with the control non-treated cells after 24 h. The data reinforces that the isolated CFCS is not cytotoxic to the tested mammalian cells.

### 3.3. In Vitro Antiviral Activity of CFCS Was Evaluated Using MDCK Cells

**Figure 2:**
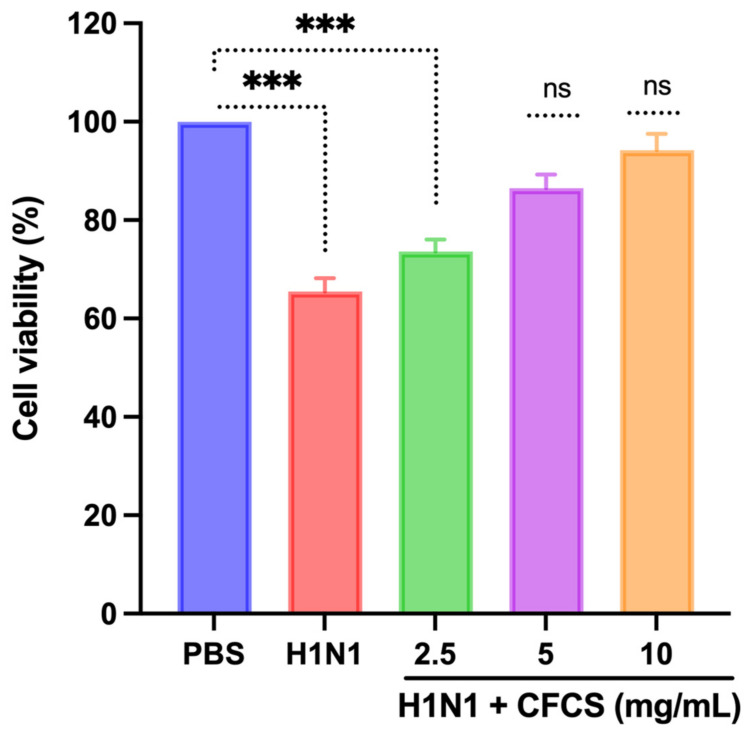
Efficacy of CFCS against the cytopathic effect of the H1N1 virus. The percentage of cell viability was determined using an MTT assay. The experiments were performed in triplicate, presenting the results as mean ± SE values. The statistical significance was evaluated by comparing the cell viability percentage of treatment groups with control (PBS only). *p*-values: *** < 0.001. ns = non-significant.

A potent antiviral effect of *L. plantarum* KAU007 was examined in MDCK cells against influenza A virus H1N1. The results underpin that H1N1 viral dose (10^6.5^ EID_50_/0.1 mL) induced CPE in MDCK cells. As a result, it reduced the number of viable MDCK cells (*p* < 0.001) compared to non-infected MDCK control cell viability. Nevertheless, MDCK cells treated with CFCS (5 and 10 mg/mL) isolated from *L. plantarum* KAU007 showed no CPE even after 72 h of the viral injection compared to control (non-treated) and infected cells. A significant (*p* < 0.001) difference in the cell viability was still observed even after 2.5 mg/mL CFCS treatment to H1N1 infected cells. Therefore, *L. plantarum* KAU007 may be considered an effective inhibitor of CPE in MDCK cells. Serial dilutions of CFCS (5 and 10 mg/mL) were also able to control the CPE in MDCK cells, as shown in Figure 2. Similar results were also reported by Rather et al. [16], where they observed that *L. plantarum* isolated from Kimchi also has the antiviral activity. One of our previous studies recently showed that *L. plantarum* inhibits SARS-CoV [31].

### 3.4. Antiviral Activity of CFCS Was Evaluated Using SPF Embryonated Eggs and Hemagglutination Assay

**Figure 3:**
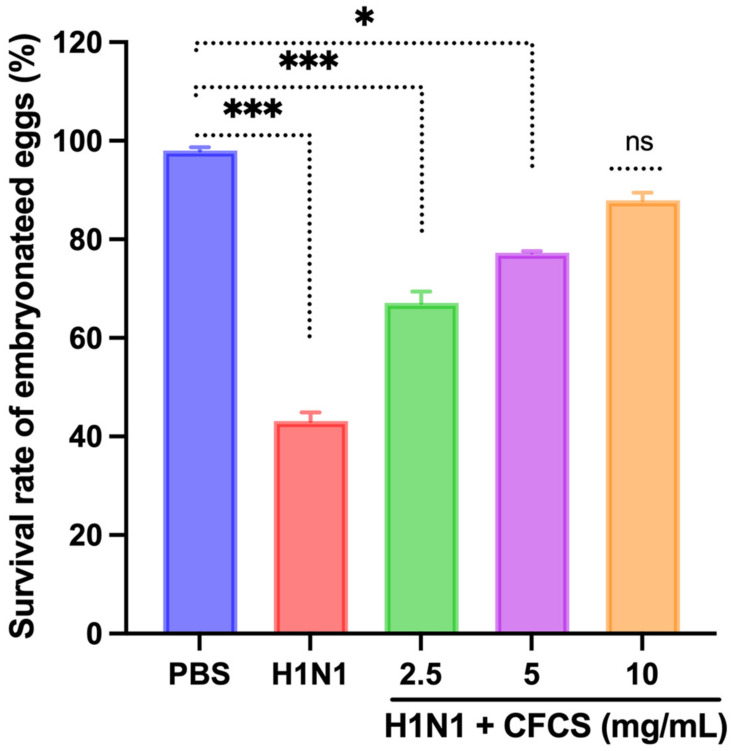
Effect of CFCS on survival percentage of H1N1 virus-challenged embryonated eggs. The experiments were performed in triplicate, presenting the results as mean ± SE values. The statistical significance was evaluated by comparing the survival rate of embryonated eggs in treatment groups with control (PBS only). Results were statistically evaluated compared to the control group (PBS only). *p*-values: * < 0.05, *** < 0.001, ns = non-significant.

**Figure 4:**
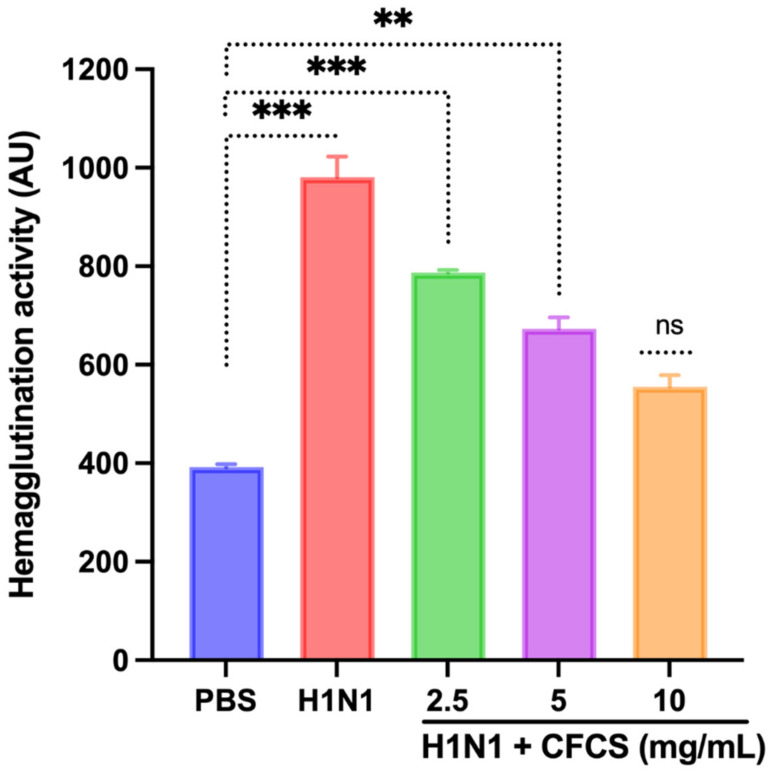
Effect of CFCS on hemagglutination activity. The experiments were performed in triplicate, presenting the results as mean ± SE values. The statistical significance was evaluated by comparing hemagglutination activity of treatment groups with control (PBS only). *p*-values: ** < 0.01, *** < 0.001, ns = non-significant.

A viral dose of 10^6.5^ EID_50_/0.1 mL was inoculated into embryonated SPF eggs, and CFCS (2.5, 5, and 10 mg/mL) of *L. plantarum* KAU007 was similarly used. Based on our results, we found that 10 mg/mL CFCS promoted the best survival rate for embryonated eggs (Figure 3). The hemagglutination assay (HA) was used to screen hemagglutinating agents in egg culture and amniotic fluid collected from embryonated eggs against viruses. *L. plantarum* KAU007 was examined to confirm its antiviral properties and detect live and inactivated viruses. Using a concentration of 5 mg/mL and 10 mg/mL of CFCS, we found that CFCS revoked viral infection completely (Figure 4). However, 2.5 mg/mL was less effective against the H1N1 virus infection. Similarly, the results of Sunmola et al. have proved that *L. plantarum* AA09a can effectively reduce viral infectivity [50].

### 3.5. Estimation of Cytokines by ELISA

**Figure 5:**
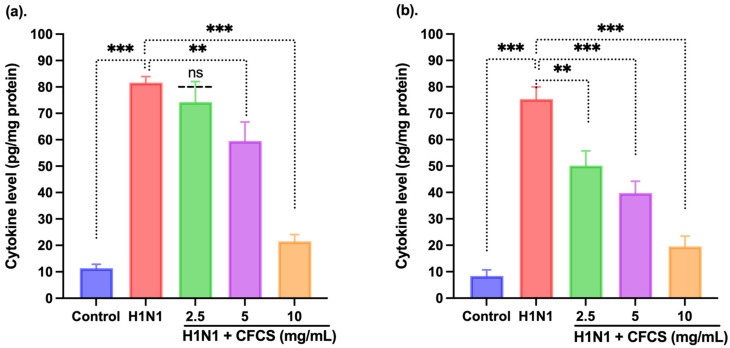
Effect of CFCS treatment on interleukin levels in H1N1 virus-challenged mammalian kidney cells. (**a**) IFN-γ and (**b**) IL-6. The experiments were performed in triplicate, presenting the results as mean ± SE values. The statistical significance was evaluated by comparing cytokine levels of control and treatment groups with the H1N1 only challenged group. *p*-values: ** < 0.01, *** < 0.001, ns = non-significant.

Several pro-inflammatory cytokines, not limited to IFN-γ and IL-6, play a major role in the development and regulation of inflammation. Therefore, we tested the effect of CFCS on the expression levels of these two cytokines. It was found that the expression levels of both cytokines significantly decreased. However, a significant increase in IFN-γ was observed in H1N1 (10^6.5^ EID_50_/0.1 mL) infected cells (81.64 ± 4.23 pg/mL) when compared to the control cells having an amount of 11.4 ± 4.22 pg/mL (*p* < 0.001). Moreover, CFCS at 5 and 10 mg/mL significantly decreased the level of IFN-γ in a dose-dependent manner, *p* < 0.001 and *p* < 0.001, respectively, as depicted in Figure 5. However, 2.5 mg/mL CFCS treatment showed non-significant changes compared to the H1N1 infected cells. In addition, the interplay between IFN-γ and IL-6 impacting the inflammatory response was observed on the H1N1 infected cells, as shown in Figure 5. By using ELISA, we found that the total IL-6 level in infected cells (75.38 ± 2.19 pg/mL) was also significantly higher than that of control cells (8.4 ± 2.14 pg/mL) (*p* < 0.001). However, a significant decrease in the level of IL-6 was observed (*p* < 0.01, *p* < 0.01, and *p* < 0.001) when treated with 2.5, 5, and 10 mg/mL of CFCS, respectively, compared to H1N1 infected cells. Studies conducted by other researchers have also shown that *L. plantarum* could decrease pro-inflammatory cytokines, such as TNF-α, IL-6, and IL-8 [50]. Additionally, *L. plantarum* IS-10506 was also found to regulate the immune system by decreasing IL-17, IL-4, and IFN-γ levels in serum of atopic dermatitis [51]. Similar results were observed when the CFCS of *L. plantarum* was treated to H1N1 infected cells.

Probiotics have a long evolutionary history of use in fermented foods and dairy for their beneficial effects on the host [14,17,19,20,21,25,27,45,46]. There has been a growing interest in probiotics as nutraceuticals from the last few decades. Besides, thousands of scientific studies reinforce the efficacy of probiotics against various diseases not limited to bacterial, viral, and fungal infections. As a result of their antimicrobial and probiotic properties, LAB has attracted increased attention in recent years, implying promising alternatives to chemically synthesized drugs [52,53,54]. Several different LAB strains were isolated from different camel milk samples, and their antiviral potential was tested against the H1N1 influenza virus. Among those 10 LAB strains, *L. plantarum* KAU007 was identified to be a highly potent strain and showed the strongest antiviral activity against H1N1 influenza. The CFCS isolated from *L. plantarum* KAU007 did not show any cytotoxicity against MDCK cells. These results support that the isolated *L. plantarum* KAU007 can be considered a safe candidate that may not affect the host’s cells. The in vitro antiviral activity assays against the MDCK cell line show that *L. plantarum* KAU007 has very strong antiviral activity against the H1N1 virus. Of note, 10 mg/mL concentration of CFCS of *L. plantarum* KAU007 was the most effective dose against the H1N1 virus. Similar results were also observed using embryonated SPF eggs, and the antiviral activity was seen to be the most at 10 mg/mL concentration of CFCS. Therefore, based on our results, it could be concluded that *L. plantarum* KAU007 may be a potent probiotic candidate found in camel milk which possesses anti-influenza prevention activity. The study needs to investigate the mechanism of action and target other influenza strains to develop an effective therapeutic candidate as a preventive measure against respiratory illness caused by influenza viruses.

## 4. Conclusions

The current study underpins the importance of camel milk and its beneficial probiotic isolates. To the best of our knowledge, this is for the first time that a LAB isolate from camel milk was evaluated against H1N1. Camel milk has the highest probiotic potential besides being rich in essential nutrients. However, the type of LAB present in camel milk varies greatly from region to region. Therefore, this study reinforces that KAU007 may be a novel antiviral probiotic strain to prevent influenza infection. This is a preliminary study; further in vivo investigations are necessary to assess the strength of the isolate against H1N1 and other strains.

## Data Availability

The data available has been presented in the paper.
